# Recombinant *Bacillus subtilis* spores expressing cholera toxin B and ovalbumin prevent ovalbumin-specific food allergy in mice by upregulating regulatory T cells and modulating gut microbiome flora

**DOI:** 10.3389/fimmu.2026.1872151

**Published:** 2026-07-22

**Authors:** Zhile Xiong, Xiuju Liu, Xiaoying Deng, Chao Zhang, Kaiyue Yang, Zhimin Zhao, Tongyan Ding, Shuyan Liu, Zhenwen Zhou

**Affiliations:** 1 Clinical Laboratory, Key Laboratory of Bacterial Resistance and Prevention, Medical Research Institute of Maternal and Child, Affiliated Shenzhen Women and Children's Hospital (Longgang) of Shantou University Medical College (Longgang District Maternity & Child Healthcare Hospital of Shenzhen City), Shenzhen, China; 2 Clinical Laboratory, Guangzhou Women and Children's Medical Center, Guangzhou Medical University, Guangzhou, China; 3 Charité – Universitätsmedizin Berlin, Corporate Member of Freie Universität Berlin and Humboldt- Universität zu Berlin, Institute of Microbiology, Infectious Diseases and Immunology, Berlin, Germany; 4 Department of Gastrointestinal Surgery, Department of General Surgery, Guangdong Provincial People's Hospital (Guangdong Academy of Medical Sciences), Southern Medical University, Guangzhou, China

**Keywords:** *Bacillus subtilis*, fecal microbiota transplant, microbiome, oral tolerance, OVA-food allergy, Treg

## Abstract

**Background:**

Although oral immunotherapy has shown clinical efficacy in treating food allergies, its broader implementation is constrained by the occurrence of adverse effects. Consequently, inducing allergen-specific immune tolerance during early life can be a preventive strategy to reduce the development of food allergy.

**Objective:**

Here, we developed a novel fusion protein cholera toxin B (CTB)-ovalbumin (OVA) expressed on *Bacillus subtilis* (*B.s*-CotC-CTB-OVA) spore surface and investigated whether *B.s*-CotC-CTB-OVA spores prevent OVA-induced food allergy in a mouse model and explored the potential underlying mechanisms.

**Method:**

Sodium dodecyl sulfate–polyacrylamide gel electrophoresis (SDS-PAGE) and Western blot were used to confirm that CTB-OVA was expressed on *B. subtilis* spores. Female BALB/c mice were orally administered with *B.s*-CotC-CTB-OVA spores and *B. subtilis* spore control (*B.s*-CotC and *B.s*-CotC-CTB) for 4 weeks. Then, sensitization and challenge with OVA were performed on mice. Fecal OVA-secretory IgA (sIgA) and serum OVA-IgE, IgG1, and IgG2a levels were measured by enzyme-linked immunosorbent assay (ELISA). The gut microbiome was analyzed by 16S rDNA sequencing. After challenge, diarrhea score, anaphylactic reactions score, splenocyte interleukin (IL)‐10, IL‐4, and interferon‐γ (IFN‐γ), and Treg levels were measured. mRNA of IL-10, IL-4, IFN‐γ, and Foxp3 were measured. Fecal microbiota transplant (FMT) was used to explore the mechanisms of microbiome in *B. subtilis* on food allergy.

**Results:**

Recombinant CTB-OVA was successfully expressed on the surface of *B. subtilis*. Oral administration of *B.s*-CotC-CTB-OVA can increase fecal OVA-sIgA, alleviate food allergy symptoms, and decrease serum OVA-IgE in mice with significance (*p* < 0.05). Moreover, oral administration of *B.s*-CotC-CTB-OVA can significantly reduce serum OVA-IgG1, OVA-IgG2, IL-4, spleen mast cells, and eosinophil levels and significantly increase serum IL-10 and Treg levels (*p* < 0.05). Additionally, microbiome analysis shows that oral administration of *B.s*-CotC-CTB-OVA can significantly increase the relative abundance of *Muribaculaceae* and significantly decrease the relative abundance of *Alistipes.* FMT partially reproduced the reduction in serum OVA-specific IgE, but did not significantly improve allergic symptom or diarrhea scores, suggesting that gut microbiota alterations may partially contribute to the immunological effects of B.s-CotC-CTB-OVA.

**Conclusion:**

These findings suggest that *B.s*-CotC-CTB-OVA spores may serve as a preventive oral antigen-delivery strategy to promote antigen-specific immune regulation and partially modulate microbiota-associated immune responses in OVA-induced food allergy.

## Introduction

Food allergies have emerged as a major public health concern, with current estimates indicating that they affect up to 10% of the global population, particularly in industrialized countries (1). Recent studies have introduced innovative methods for the prevention and treatment of food allergies. Oral immunotherapy (OIT) has emerged as a promising strategy, involving the incremental exposure to specific allergens—such as peanuts, milk, and eggs—with the aim of desensitizing patients and attenuating the severity of allergic reactions. The initial OIT pharmaceutical, Palforzia, has been approved by the U.S. Food and Drug Administration (FDA) with documented efficacy in mitigating the frequency and intensity of peanut-induced allergic reactions by modulating the Th2 immune response (2). However, approximately 10%–20% of patients discontinue Palforzia treatment because of adverse events, most commonly gastrointestinal symptoms. Moreover, long-term sustained unresponsiveness is not achieved in the majority of patients (3).

Studies have demonstrated that the early introduction of low-level allergens before immunization has been suggested to aid in reducing the likelihood of allergic disease onset in genetically susceptible individuals (4, 5). Seidel et al. discovered that low doses of contact allergens have the capacity to impede the progression of TC1-mediated contact hypersensitivity of the epicutaneous variety (6). Moreover, Yeh et al. have demonstrated that the intranasal administration of a protein antigen to mice prior to immunization has been shown to induce immunological tolerance and suppress allergic airway inflammation (7). These findings suggest a common underlying mechanism: during early life, especially before the immune system fully matures, low-dose antigen exposure via mucosal surfaces can promote immune tolerance rather than sensitization, and this “early education” of the immune system may play a crucial role in regulating long-term immune responses and offers a promising strategy for the prevention of allergic diseases. Moreover, recently, the composition of the gut microbiome has been reported to be associated with the development of food allergy and asthma, paving the way for potential probiotic-based treatments (8–10).

*Bacillus subtilis* has been proven to be a probiotic and food additive that is beneficial to human health. It is capable of forming spores and tolerating various adverse environmental conditions, including high temperatures, acidic pH, oxidative stress, and the gastrointestinal environment (11, 12). This has led to its emergence as a promising novel biological vector for delivering proteins to the human gastrointestinal tract (13). In a previous study, we successfully constructed recombinant *B. subtilis* spores expressing cholera toxin B (CTB) and neutrophil-activating protein (NAP) and suppressing peanut allergy and milk allergy with decreasing serum IgE and increasing fecal IgA (14, 15). Ovalbumin (OVA), a major protein in egg white, is one of the most common food allergens. CTB constitutes a non-toxic component of the *Vibrio cholerae* holotoxin, which, in autoimmune diseases, has been demonstrated to promote tolerance to mucosal co-administered antigens. Building upon this immunomodulatory property, we developed a novel oral allergy vaccine based on *B. subtilis* spores co-expressing CTB and OVA. The goal of this strategy is to preimmunize mice during early life in order to induce immune tolerance and thereby prevent the development of OVA-induced food allergy. In addition, we analyze the underlying mechanism between CTB-OVA *B. subtilis* to microbiome and food allergy axis.

## Material and method

### Mice

Pathogen-free female BALB/C mice (6 weeks old) were obtained from the Guangdong Medical Laboratory Animal Center (Foshan, China). The mice were kept in a controlled environment and provided with a diet and water that were free of OVA and certain pathogens. All procedures were carried out in compliance with the Ethics Committee of Guangzhou Women and Children’s Medical Center (registration no. 108B00). Mice were randomly assigned to experimental groups before treatment. Unless otherwise indicated, the main food allergy experiment included *n* = 4 mice in the naïve group and *n* = 6 mice in each OVA-sensitized group. The FMT experiment included *n* = 4 mice in the naïve group and *n* = 5 mice in each OVA-sensitized group. For fecal microbiota analysis, five mice per group were used for 16S rRNA gene sequencing.

### The construction of *B.s*-CotC-CTB-OVA spores

The approach employed in constructing the CTB-OVA gene is detailed in **Supplementary Figure 1** and described below. OVA DNA (1,169 bp) was amplified by polymerase chain reaction (PCR) using the synthesized OVA DNA (GenScript, Piscataway, NJ) as template with the following designed primers: forward primer: 5′-AACGAATTCATGGGCTCCATCGGTGC-3′ with a EcoR I site (underlined), reverse primer: 5′-AAAGCGGCCGCTTAAGGGGAAACACATCTG-3′ with a Not I site (underlined). The PCR conditions were performed as follows: 94 °C for 5 min followed by 30 cycles of 94 °C for 30 s, 59 °C for 30 s, and 72 °C 60 s and a final extension at 72 °C for 5 min. The PCR products were digested with EcoR I and Not I and cloned into III and ligated to the 3′ end of the CTB gene in pET 28-CTB plasmid constructed before (16) and transformed to *Escherichia coli* BL21. CTB-OVA DNA (1,482 bp) was amplified by using the pET28-CTB-OVA plasmid as template with PCR primers: forward primer: 5′-CGGTCTAGAGACACCTCAAAATATTACTGATT-3′ with an Xba I site (underlined), reverse primer: 5′-CGCAAGCTTTTAAGGGGAAACACATCTG-3′ with a Hind III site (underlined). The purified PCR product was double digested by Xba I/Hind III and ligated to the 3′ end of the CotC gene in pUS186-CotC plasmid constructed before (17) and transformed into *B. subtilis* WB600 by the standard competent cell method (17, 18) and confirmed by Xba I/Hind III double enzyme digestion and DNA sequencing.

### Preparation of recombinant *B. subtilis* spores

*B. subtilis* spores expressing spore coat protein C (CotC) (*B.s*-CotC), CotC-CTB (*B.s*-CotC-CTB), and CotC-CTB-OVA (*B.s*-CotC-CTB-OVA) were cultivated in Difco sporulation medium (DSM; BD Biosciences, San Diego, CA, USA). Following 24 h of sporulation induction, spores were isolated using methods outlined in earlier studies (15). Briefly, the harvested spores were centrifuged at 8,000 × *g* and were subsequently incubated with 4 mg/mL lysozyme at 35 °C for 30 min to lyse the remaining mother cells. To eliminate residual cellular material, the spores were washed sequentially with 1 M NaCl, 1 M KCl, and distilled water, then suspended in sterile water at 68 °C for 1 h. Outer coat proteins were obtained via centrifugation, and the recombinant spore-associated proteins were extracted using a buffer containing SDS and dithiothreitol (DTT). Expression of CTB-OVA fusion proteins on the *B. subtilis* spore surface was validated by sodium dodecyl sulfate–polyacrylamide gel electrophoresis (SDS-PAGE) and Western blotting, as previously reported (14, 16).

### OVA allergy model and spore treatment

**Figure 1:**
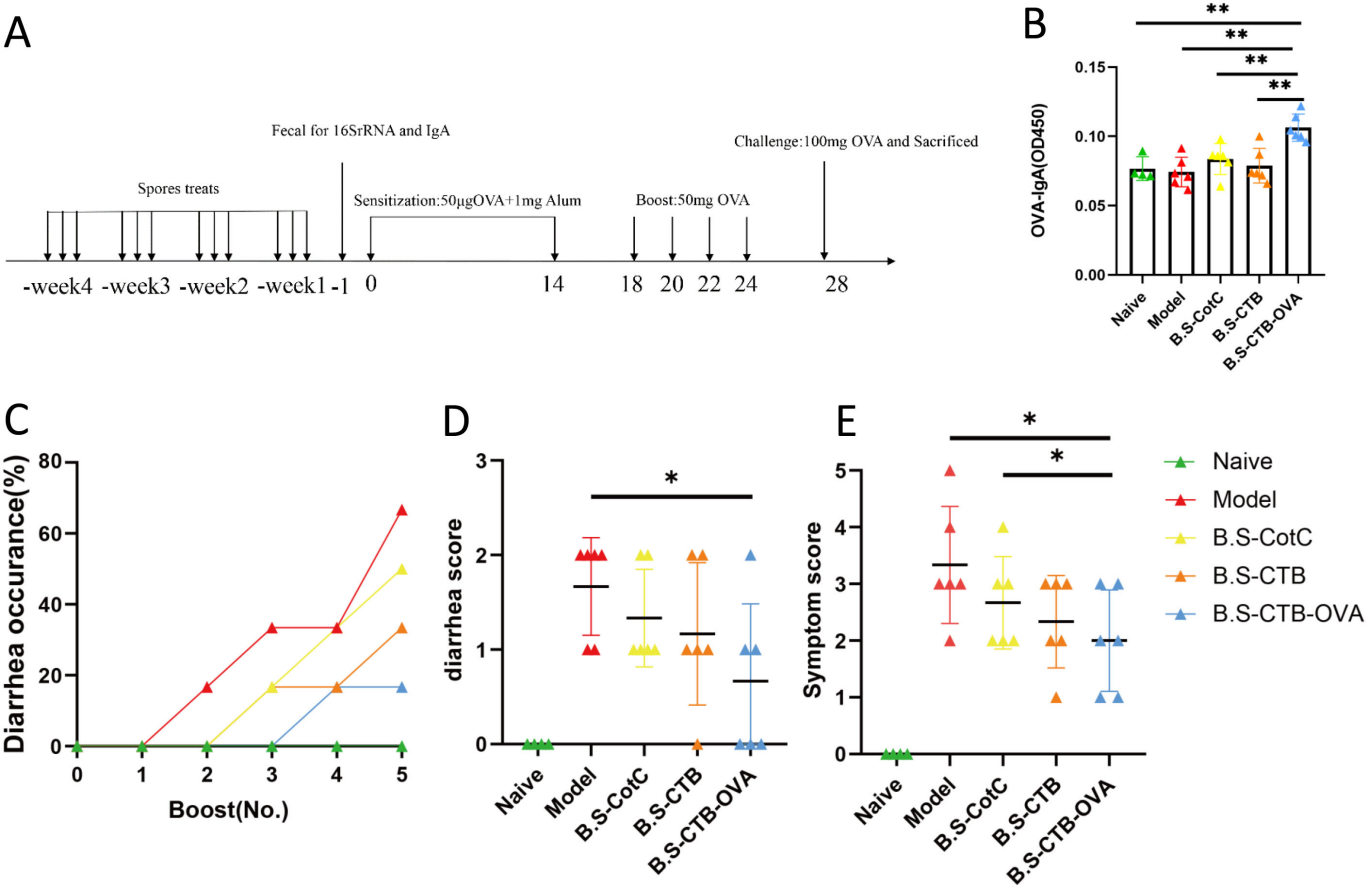
Oral delivery of B.s-CotC-CTB-OVA spores enhances fecal sIgA production and alleviates OVA-induced allergic responses. **(A)** Experimental protocol for *B. subtilis* spore administration, OVA sensitization, OVA boosts, and final OVA challenge. **(B)** Fecal OVA-specific sIgA levels were measured by ELISA before OVA sensitization. **(C)** Diarrhea occurrence was recorded after repeated OVA boosts. **(D)** Diarrhea scores were evaluated after OVA challenge. **(E)** Allergic symptom scores were evaluated after OVA challenge. Statistical analyses were performed as described in the Methods section. Each symbol represents an individual mouse. *n* = 4 for the naïve group and *n* = 6 for the other groups. *p < 0.05, **p < 0.01, ***p < 0.001.

The strategy for the treatment of *B. subtilis* spores to mice and the establishment of an OVA allergy model with OVA sensitization, boosting, and challenge is shown in **Figure 1A**. Mice were fasted for at least 6 h prior to oral administration. A total of 1.0 × 10 (9) *B.s*-CotC spores, 1.0 × 10 (9) *B.s*-CotC-CTB spores, and 1.0 × 10 (9) *B.s*-CotC-CTB-OVA spores or phosphate-buffered saline (PBS) were delivered to the mice by oral administration three times each week for 4 weeks. All mice, except mice in the PBS group, were sensitized by intraperitoneal (i.p.) injections of 50 μg of OVA (A-5503; Sigma-Aldrich, St Louis, Missouri, USA) mixed with 1 mg of aluminum adjuvant on day 1 and day 14. Following sensitization, mice were boosted with OVA (50 mg/mouse) in 0.25 mL of saline intragastrically four times every other day on days 18 until 24. Lastly, mice were challenged with 500 μL of 100 mg/mL OVA on day 28.

### Assessment of OVA food allergy symptoms and diarrhea score

Food allergy symptom score was evaluated 15–45 min after boost and challenge according to the scoring scheme previously described (19, 20): 0—no observable symptoms; 1—scratching or rubbing around the head and snout; 2—swelling around the eyes or snout, piloerection, reduced mobility, and/or elevated breathing rate; 3—audible wheezing, difficulty breathing, and bluish discoloration near the mouth or tail; 4—unresponsiveness to stimulation, tremors, or convulsions; and 5—death. The diarrhea score was defined as previously described and as follows: 0—normal stool, 1—soft stool, and 2—loose or liquid stool (19, 21). An illustration of the scoring system is provided in **Supplementary Figure 2**.

### Measurement of OVA-specific serum and mucosal antibodies

OVA-IgE antibody titers in sera and IgA antibody titers in fecal microbiota were measured by enzyme-linked immunosorbent assay (ELISA) as previously described (16). In brief, 96-well ELISA plates were pre-coated with OVA solution (5 μg/mL) and stored at 4°C overnight. On the following day, plates were washed five times using PBST and blocked with 10% fetal bovine serum (FBS; Ruite Biotechnology Co., Guangzhou, China) at 37°C for 2 h. After blocking, 100 μL of fecal supernatants or serum samples was added to the wells and incubated overnight at 4°C. On the second day, horseradish peroxidase (HRP)-conjugated goat anti-mouse antibodies targeting secretory IgA (sIgA) or IgE (1:5,000; Southern Biotech, USA) were applied and incubated at 37°C for 90 min. After another five PBST washes, 100 μL of TMB substrate followed by 50 μL of stop solution (both from KHB, Shanghai, China) were sequentially added; 450 nm was used to measure the optical density.

### Cell culture and cytokine measurements

Splenocytes were resuspended in complete RPMI-1640 medium (supplemented with 10% FBS and 1% penicillin–streptomycin) at a final concentration of 8 × 10^6^ cells/mL. A total of 250 μL of cell suspension was seeded into each well of a 24-well plate, followed by the addition of 250 μL of OVA solution (400 μg/mL in PBS). For stimulation controls, cells were cultured in parallel with phytohemagglutinin (PHA, 5 μg/mL in complete medium) as a positive control, and complete medium alone served as a negative control. After a 72-h incubation, culture supernatants were collected for cytokine quantification. The levels of interleukin (IL)-4, IL-10, and IFN-γ in medium were measured by commercial ELISA kits (BD Biosciences).

### mRNA expression in the jejunum

Total RNA extraction and quantitative real-time PCR (qPCR) analysis of Foxp3, IL-4, IL-10, and IFN-γ expression were performed as previously described (15). Briefly, jejunal tissues were collected and homogenized, and total RNA was extracted using an RNA Extraction Kit (Tiangen, Beijing, China) according to the manufacturer’s instructions. Purified RNA was reverse-transcribed into cDNA using a Superscript II reverse transcription kit (TransGen, Beijing, China). Quantitative real-time PCR was subsequently performed using SYBR Green qPCR Master Mix (DBI Bioscience, Shanghai, China) on a real-time PCR detection system. Relative mRNA expression levels were normalized to Gapdh and calculated using the 2^−δδCT^ method. Primer sequences were listed as follows: GAPDH forward primer: 5′-TGTAGACCATGTAGTTGAGGTCA-3′, reverse primer: 5′-AGGTCGGTGTGAACGGATTTG-3′; Foxp3 forward primer: 5′-CCCAGGAAAGACAGCAACCTT-3′, reverse primer: 5′-TTCTCACAACCAGGCCACTTG-3′; IL-4 forward primer: 5′-TACCAGGAGCCATATCCACGGATG-3′, reverse primer: 5′-TGTGGTGTTCTTCGTTGCTGTGAG-3′; IL-10 forward primer: 5’-CGGGAAGACAATAACTGCACCC-3’, reverse primer: 5’-CGGTTAGCAGTATGTTGTCCAGC-3’; IFN-γ forward primer: 5′-GAGCCAGATTATCTCTTTCTACC-3′, reverse primer: 5′-TTGTTGACCTCAAACTTGG-3′.

### Measurement of spleen Tregs, mast cells, and eosinophils

Spleens were collected after the final OVA challenge. Tissues were rinsed with PBS and mechanically dissociated through a 70-μm nylon cell strainer to obtain single-cell suspensions. Red blood cells were removed using red blood cell lysis buffer, and the remaining cells were washed and resuspended in staining buffer. For each sample, 1 × 10^6^ cells were used for antibody staining.

Separate antibody panels were used for the analysis of eosinophils, mast cells, and Tregs. For eosinophil analysis, cells were stained with anti-CD45-APC-Cy7, anti-CD11b-BV510, anti-Siglec-F-PE, and propidium iodide (PI) for dead-cell exclusion. Eosinophils were identified as CD11b^+^Siglec-F^+^ cells within the CD45^+^PI^-^ live leukocyte population.

For mast cell analysis, cells were stained with anti-CD45-APC-Cy7, anti-IgE-BV510, anti-c-kit-PE, and PI. Mast cells were identified as c-kit^+^IgE^+^ cells within the CD45^+^PI^-^ live single-cell population.

For Treg analysis, cells were stained with anti-CD4-APC, anti-CD8-APC-Cy7, anti-CD3-BV785, Zombie-BV510 viability dye, anti-CD25-PE, and anti-Foxp3-BV421. Tregs were identified as CD25^+^Foxp3^+^ cells within the CD4^+^ T-cell population.

All antibodies were purchased from BioLegend (San Diego, CA, USA). Stained cells were acquired using a FACSAria SORP flow cytometer (BD Biosciences, San Jose, CA, USA) and analyzed with FlowJo software. The frequencies of eosinophils, mast cells, and Tregs were calculated as percentages of the corresponding parent gates, as indicated in the figure legends and **Supplementary Figures 3**–**5**.

### 16S rRNA microbiome sequencing

Fecal microbiome DNA from the mice feces was extracted with a DNeasy PowerSoil kit (Qiagen, Germany) according to the manufacturer’s protocol. The bacterial 16S rRNA V4 variable region of extracted DNA was amplified with primers V4-515F (GTGCCAGCMGCCGCGGTAA) and V4-806R (GGACTACHVGGGTWTCTAAT). The PCR was prepared in a total reaction volume of 25 μL with 0.5 μM V4 forward and reverse primers, 200 μM deoxynucleotide triphosphates (dNTPs), and 0.25 μL of TaKaRa Premix Taq^®^ Version 2.0 (TaKaRa Biotechnology Co., Dalian, China). The PCR was performed as follows: 94 °C for 5 min followed by 30 cycles of 94 °C for 30 s, 52 °C for 30 s, 72 °C for 60 s, and a final extension at 72°C for 5 min. Then, the libraries were sequenced on the Illumina Nova 6000 with a paired-end 250 bp (PE250) at Guangdong Magigene Biotechnology Co., Ltd. Guangzhou, China.

### Fecal microbiota transplant

Fecal slurry for fecal microbiota transplant (FMT) was prepared as previously described (22, 23). Briefly, fresh feces (300 mg) were collected daily after oral administration of the *B. subtilis* spores (*B.s*-CotC and *B.s*-CotC-CTB-OVA) and naïve group. Mice used for microbiome analysis were age- and sex-matched female BALB/c mice obtained from the same supplier. Animals were randomly assigned to treatment groups and maintained under the same specific pathogen-free conditions, with identical diet, water, bedding, and light/dark cycles. Fresh fecal pellets were collected individually from each mouse at the same time point, immediately frozen in liquid nitrogen, and stored at −80 °C until DNA extraction. Mice were not specifically selected as littermates, and treatment groups were not co-housed.

**Figure 2:**
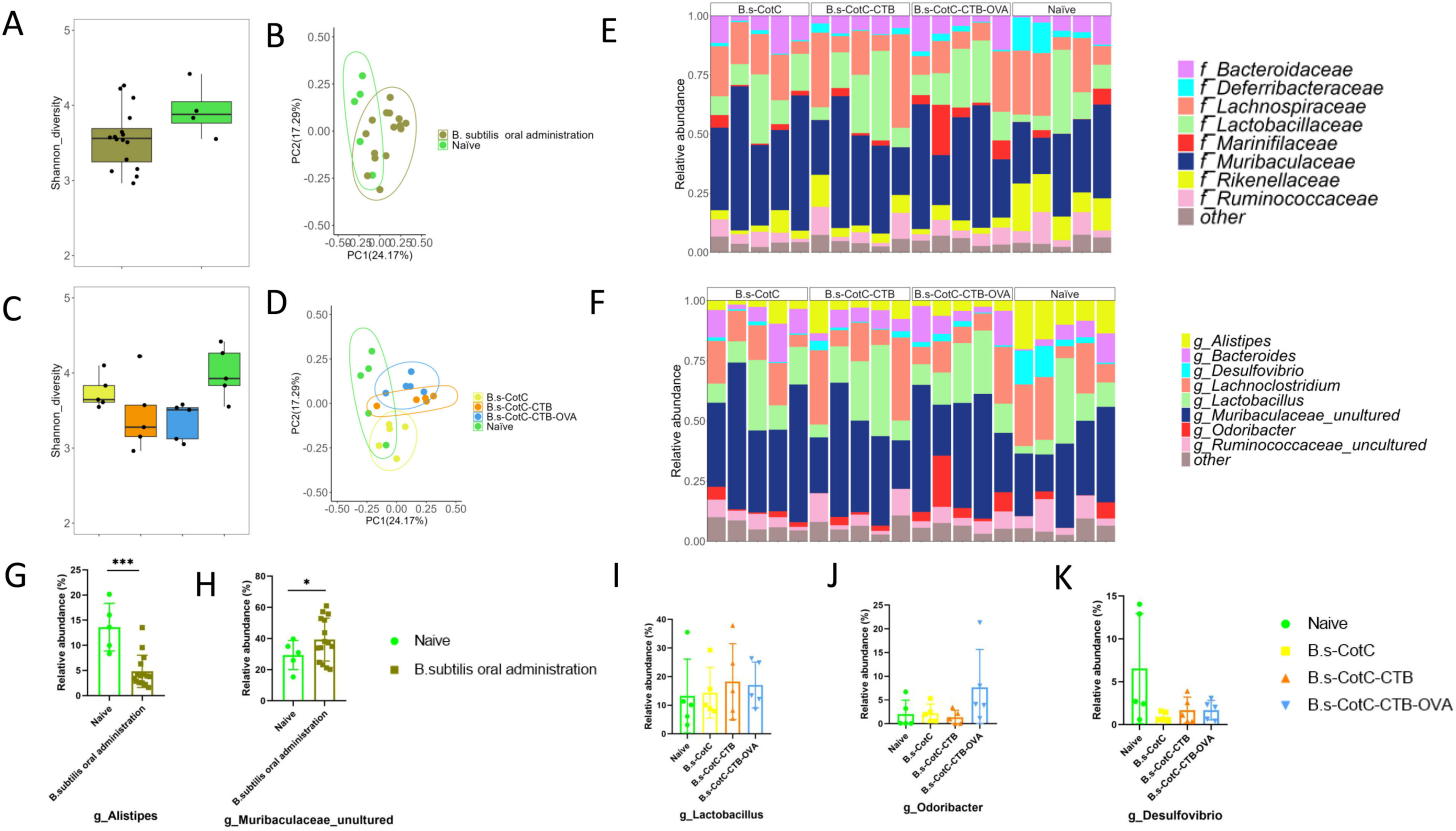
Alterations in gut microbiota composition following oral administration of B.s-CotC-CTB-OVA spores. **(A)** Shannon diversity indices comparing mice administered *B. subtilis* spores versus the naïve group. **(B)** PCoA based on Bray–Curtis distance showing clustering of microbiota profiles between the *B. subtilis*-treated and naïve groups. **(C)** Shannon diversity analysis among groups receiving B.s-CotC, B.s-CotC-CTB, B.s-CotC-CTB-OVA, or no treatment (naïve). **(D)** PCoA plots based on Bray–Curtis distances comparing microbial communities across the same four treatment groups. **(E)** Taxonomic composition profiles of fecal microbiota in mice administered *B. subtilis* spores versus naïve controls. **(F)** Microbial community structure analysis among mice treated with B.s-CotC, B.s-CotC-CTB, B.s-CotC-CTB-OVA, and naïve controls. **(G, H)** Relative abundance comparisons of specific taxa between *B. subtilis*-treated and naïve mice: **(G)** *Alistipes* and **(H)** *Muribaculaceae*. **(I–K)** Relative abundance of selected genera among the four groups: **(I)** *Lactobacillus*, **(J)** *Odoribacter*, and **(K)** *Desulfovibrio*. Fecal microbiota analysis was performed using samples from five mice per group. Statistical comparisons of α-diversity and selected taxa were performed using non-parametric tests. β-diversity was assessed by PERMANOVA based on Bray–Curtis distances. Multiple-comparison correction was applied where applicable. *p < 0.05, **p < 0.01, ***p < 0.001.

To prepare fecal slurry for FMT, fecal pellets were suspended in sterile saline (3 mL), followed by a 5-min vortex and centrifugation at 1,000 × *g* for 3 min. The fecal slurry was prepared within 10 min on the same day to minimize changes in gut microbial composition. The fecal suspension was passed through sterile gauze or a 70-μm cell strainer to remove large debris. The strategy for the FMT of mice and the establishment of an OVA allergy model with OVA sensitization, boosting, and challenge is shown in **Figure 2A**. Briefly, a combination of antibiotics including vancomycin, ampicillin, and metronidazole was orally administered to mice from day −12 to day −8. From day −7 to day −1, the recipient mice were transplanted with 0.2 mL of fecal slurry described above across to the different group.

### Bioinformatic analysis

FASTQ files from Illumina Nova 6000 sequencing were analyzed according to the MOTHUR MiSeq SOP with some modifications (MOTHUR wiki at http://mothur.org/wiki/MiSeq.SOP). Sequencing reads were taxonomically classified to the family or genus level using the SILVA 16S rRNA database. RStudio (Version R 4.2.3) was used for the analysis of data obtained from MOTHUR. α-diversity, principal coordinate analysis (PCoA) based on Bray–Curtis distances, and permutational multivariate analysis of variance (PERMANOVA) were performed in R using the ggplot2, phyloseq, and vegan packages.

### Statistical analysis

Data are presented as mean ± standard deviation (SD). For the main food allergy experiment, the sample sizes were as follows: naïve group, *n* = 4; model group, *n* = 6; *B.s*-CotC group, *n* = 6; *B.s*-CotC-CTB group, *n* = 6; and *B.s*-CotC-CTB-OVA group, *n* = 6. For the FMT experiment, the sample sizes were as follows: naïve group, *n* = 4; model group, *n* = 5; *B.s*-CotC group, *n* = 5; and *B.s*-CotC-CTB-OVA group, *n* = 5. For fecal microbiota analysis, five mice per group were used for 16S rRNA gene sequencing. The exact sample size for each experiment is also indicated in the corresponding figure legends.

For normally distributed data, comparisons between two groups were performed using an unpaired Student’s *t*-test, and comparisons among multiple groups were performed using one-way analysis of variance (ANOVA) followed by Tukey’s multiple-comparison test. For non-normally distributed data, comparisons between two groups were performed using the Wilcoxon rank-sum test, and comparisons among multiple groups were performed using the Kruskal–Wallis test followed by Dunn’s multiple-comparison test. Food allergy symptom scores and diarrhea scores were analyzed using non-parametric tests.

For microbiome analyses, α-diversity and taxonomic abundance comparisons were analyzed using non-parametric tests. For comparisons involving multiple bacterial taxa, *p*-values were adjusted for multiple testing using the Benjamini–Hochberg false discovery rate method where applicable. Differences in β-diversity among groups were assessed using PERMANOVA based on Bray–Curtis distances with the “adonis2” function in the vegan R package. A value of *p* < 0.05 was considered statistically significant. Statistical analyses were performed using GraphPad Prism version 8.0, IBM SPSS Statistics version 22.7, and R Studio with R version 4.2.3.

## Results

### Expression of CTB-OVA proteins in *B. subtilis* spores

**Figure 3:**
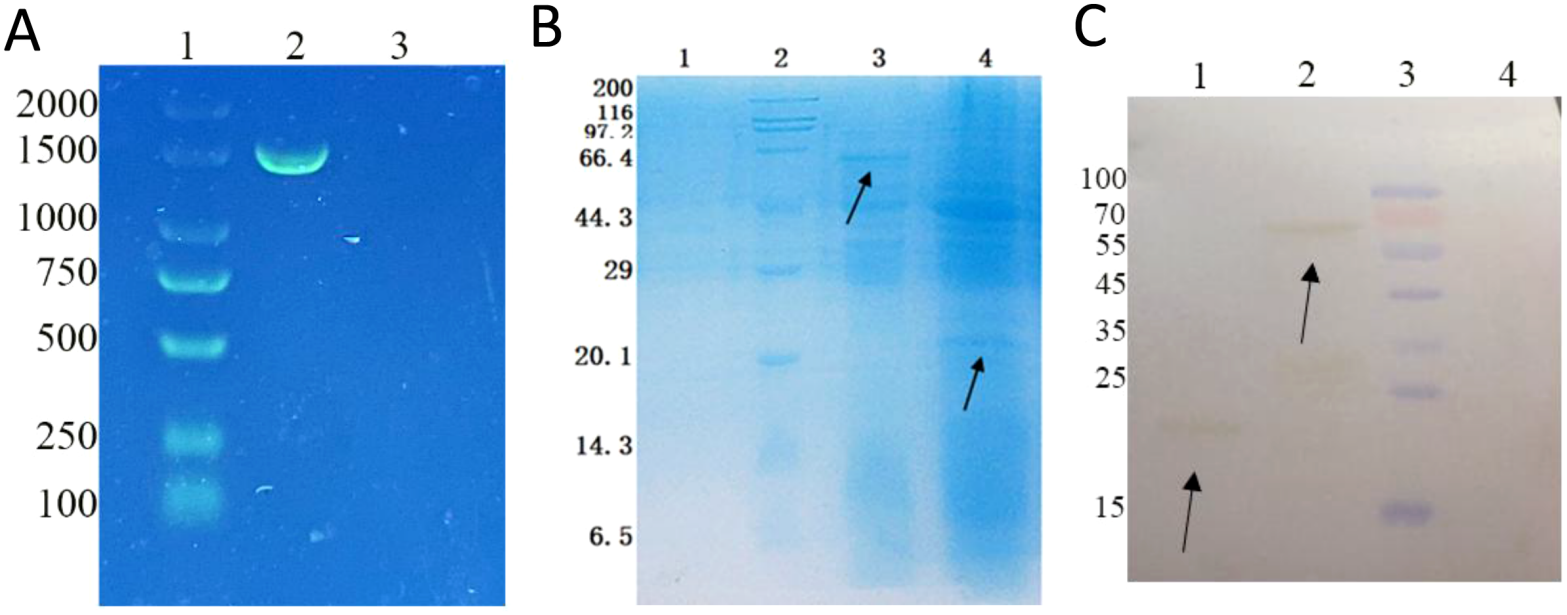
The construction of the CTB-OVA *Bacillus subtilis* spore. **(A)** PCR amplification of CTB-OVA from plasmid pUS186-CotC-CTB-OVA plasmid. Lane 1: 2 kb DNA marker; Lane 2: the target amplification fragment of CTB-OVA; Lane 3: negative control of PCR amplification of pUS186-CotC. **(B)** SDS-PAGE analysis of *B. subtilis* spore coat protein; Lane 1: *Bacillus subtilis* CotC spore; Lane 2: Protein marker ladder; Lane 3: *B. subtilis* CTB-OVA spore; Lane 4: *B. subtilis* CTB spore. **(C)** Western blot analysis of *B. subtilis* spore coat protein; Lane 1: *Bacillus subtilis* CotC spore (8.8 kDa); Lane 2: *B. subtilis* CTB-OVA spore (42.9 kDa); Lane 3: Protein marker ladder; Lane 4: *B. subtilis* CTB spore (11.6 kDa).

Previous studies have successfully shown that *B. subtilis* spores serve as an effective vehicle for the delivery of recombinant antigens to the gastrointestinal tract as a vaccine and for allergy prevention (14, 15, 24). Here, we constructed the CTB-OVA fusion protein expressed on the *B. subtilis* spores’ surfaces. First, we amplified the CTB-OVA gene and constructed the pUS186CotC-CTB-OVA plasmid. The amplification results of CTB-OVA gene from the pUS186CotC-CTB-OVA plasmid are shown in **Figure 3a** (1,482 bp). Then, we transformed the pUS186CotC-CTB-OVA plasmid into competent cell *B. subtilis* WB600. Last, we used Western blot and SDS-PAGE to detect the outer protein CTB-OVA of *B. subtilis* spores (42.9 kDa) (**Figures 3B, C**).

### Oral administration of *B.s*-CotC-CTB-OVA spores enhances fecal OVA-specific sIgA production and alleviates OVA-induced allergic responses

We further orally administered the *B.s*-CotC, *B.s*-CotC-CTB, and *B.s*-CotC-CTB-OVA spores to BALB/c mice for 4 weeks (from week −4 to week −1, a total of 28 days). On day −1, fecal samples were collected for the measurement of OVA-specific sIgA and 16S rRNA sequencing. Consistent with a previous study showing that *B. subtilis* spores can serve as an oral delivery platform to induce mucosal immune responses (14), oral administration of *B.s*-CotC-CTB-OVA spores significantly increased fecal OVA-specific sIgA levels compared with the *B.s*-CotC, *B.s*-CotC-CTB, and naïve groups (*p* < 0.01; **Figure 1B**). Mice were then sensitized and challenged with OVA according to the experimental protocol shown in **Figure 1A** (19). After each OVA boost and challenge, diarrhea scores and allergic symptom scores were recorded. Following OVA challenge, mice in the model and *B.s*-CotC groups developed typical allergic manifestations, including scratching around the nose and ears, reduced activity, and signs of respiratory distress in some animals. In contrast, mice receiving *B.s*-CotC-CTB-OVA spores exhibited milder symptoms, mainly limited to scratching around the nose and ears. Statistical analysis showed that *B.s*-CotC-CTB-OVA treatment significantly reduced allergic symptom severity compared with both the model and B.s-CotC groups (*p* < 0.05; **Figure 1E**). *B.s*-CotC-CTB-OVA treatment also significantly reduced diarrhea scores compared with the model group (*p* < 0.05; **Figure 1D**), although no significant difference in diarrhea scores was observed between the *B.s*-CotC and *B.s*-CotC-CTB-OVA groups. These results indicate that oral administration of *B.s*-CotC-CTB-OVA spores alleviates OVA-induced allergic manifestations, with a more pronounced effect on overall symptom severity than on diarrhea scores.

### Oral administration of *B.s*-CotC-CTB-OVA spores suppresses OVA-specific antibody responses and promotes IL-10 production

**Figure 4:**
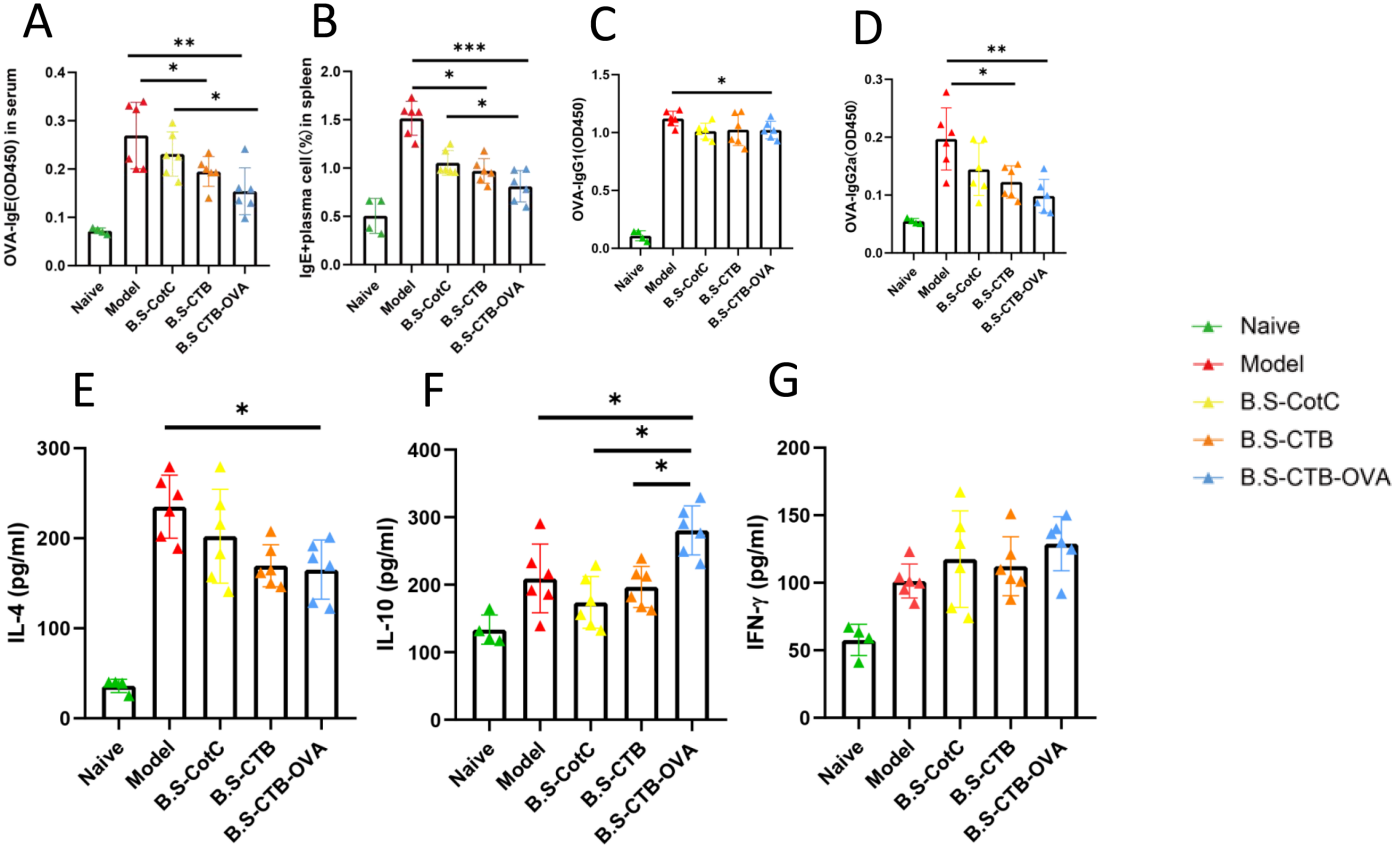
Oral administration of *B.s*-CotC-CTB-OVA spores modulates systemic immune responses by lowering OVA-specific IgE, IgG1, and IL-4 and enhancing IL-10 production. **(A)** Serum OVA-specific IgE levels were measured after OVA challenge. **(B)** Splenic IgE^+^ cells were analyzed by flow cytometry. **(C, D)** Serum OVA-specific IgG1 **(C)** and IgG2a **(D)** levels were measured by ELISA. **(E–G)** IL-4 **(E)**, IL-10 **(F)**, and IFN-γ **(G)** levels in OVA-stimulated splenocyte culture supernatants were measured by ELISA. Data are shown as mean ± SD. Each symbol represents an individual mouse. *n* = 4 for the naïve group and *n* = 6 for the other groups. Statistical analyses were performed as described in the Methods section. *p < 0.05, **p < 0.01, ***p < 0.001.

Next, we evaluated OVA-specific antibody responses after OVA challenge. Oral administration of B.s-CotC-CTB-OVA spores significantly reduced serum OVA-specific IgE levels compared with the model and B.s-CotC groups (*p* < 0.05; **Figure 4A**). Consistently, flow cytometric analysis showed a reduced frequency of splenic IgE^+^ cells in the *B.s*-CotC-CTB-OVA group (**Figure 4B**). In addition, serum OVA-specific IgG1 and IgG2a levels were significantly decreased in mice receiving *B.s*-CotC-CTB-OVA spores compared with the model and *B.s*-CotC groups (*p* < 0.05; **Figures 4C, D**). *B.s*-CotC-CTB treatment also reduced serum OVA-specific IgE, splenic IgE^+^ cell frequencies, and serum OVA-specific IgG2a levels compared with the model group (*p* < 0.05), although these effects were less pronounced than those observed in the *B.s*-CotC-CTB-OVA group. We further assessed cytokine production in OVA-stimulated splenocyte culture supernatants. Compared with the model group, *B.s*-CotC-CTB-OVA treatment significantly reduced IL-4 production and increased IL-10 production (*p* < 0.05; **Figures 4E, F**). IFN-γ levels showed an increasing trend in the *B.s*-CotC-CTB-OVA group, but the difference did not reach statistical significance among the OVA-sensitized groups (**Figure 4G**). Together, these findings suggest that *B.s*-CotC-CTB-OVA treatment suppresses OVA-specific humoral immune responses and promotes a regulatory cytokine profile.

### Oral administration of *B.s*-CotC-CTB-OVA spores reduces spleen mast cells and eosinophils and increases Treg cells

**Figure 5:**
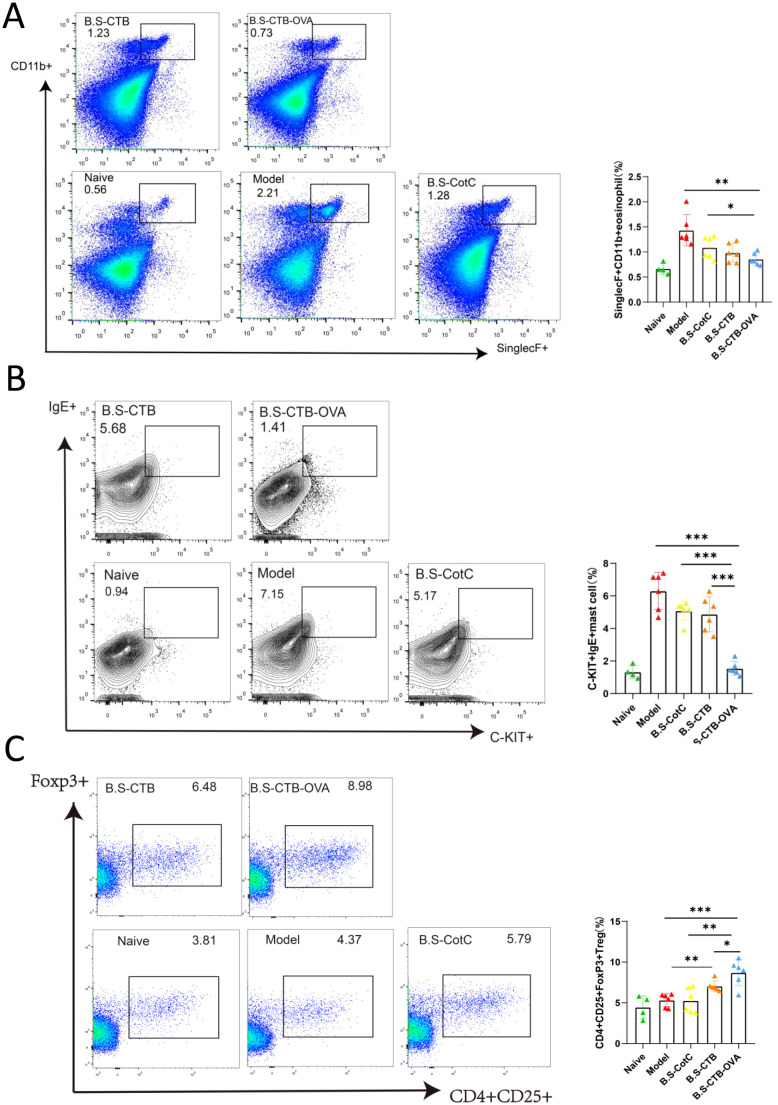
Oral delivery of B.s-CotC-CTB-OVA spores reduces splenic eosinophil and mast cell frequencies while increasing splenic Treg frequencies. **(A)** Representative flow cytometry plots and quantification of splenic eosinophils. After debris exclusion based on FSC/SSC characteristics, CD45^+^PI^-^ live leukocytes were gated, and eosinophils were identified as CD11b^+^Siglec-F^+^ cells. The frequencies shown represent percentages of the CD45^+^PI^-^ live-cell parent population. **(B)** Representative flow cytometry plots and quantification of splenic mast cells. After debris exclusion, singlet selection, and gating on CD45^+^PI^-^ live leukocytes, mast cells were identified as c-kit^+^IgE^+^ cells. The frequencies shown represent percentages of the CD45^+^PI^-^ live single-cell parent population. **(C)** Representative flow cytometry plots and quantification of splenic regulatory T cells. After lymphocyte gating, CD4^+^ T cells were selected, and Tregs were identified as CD4^+^CD25^+^Foxp3^+^ cells. The frequencies shown represent percentages of the indicated CD4^+^ T-cell parent population. Data are shown as mean ± SD. Each symbol represents an individual mouse. *n* = 4 for the naïve group and *n* = 6 for the other groups. Statistical analyses were performed as described in the Methods section. *p < 0.05, **p < 0.01, ***p < 0.001.

Flow cytometric analysis showed that oral administration of B.s-CotC-CTB-OVA spores significantly reduced the frequencies of splenic mast cells and eosinophils compared with the model and B.s-CotC groups. In parallel, splenic Treg frequencies were significantly increased in the B.s-CotC-CTB-OVA group. B.s-CotC-CTB treatment also increased splenic Treg frequencies compared with the model and B.s-CotC groups. These findings suggest that B.s-CotC-CTB-OVA treatment is associated with reduced allergic effector cell accumulation and enhanced systemic regulatory immune responses (**Figure 5**).

### Oral administration of *B.s*-CotC-CTB-OVA spores modulates jejunal immune-related gene expression

**Figure 6:**
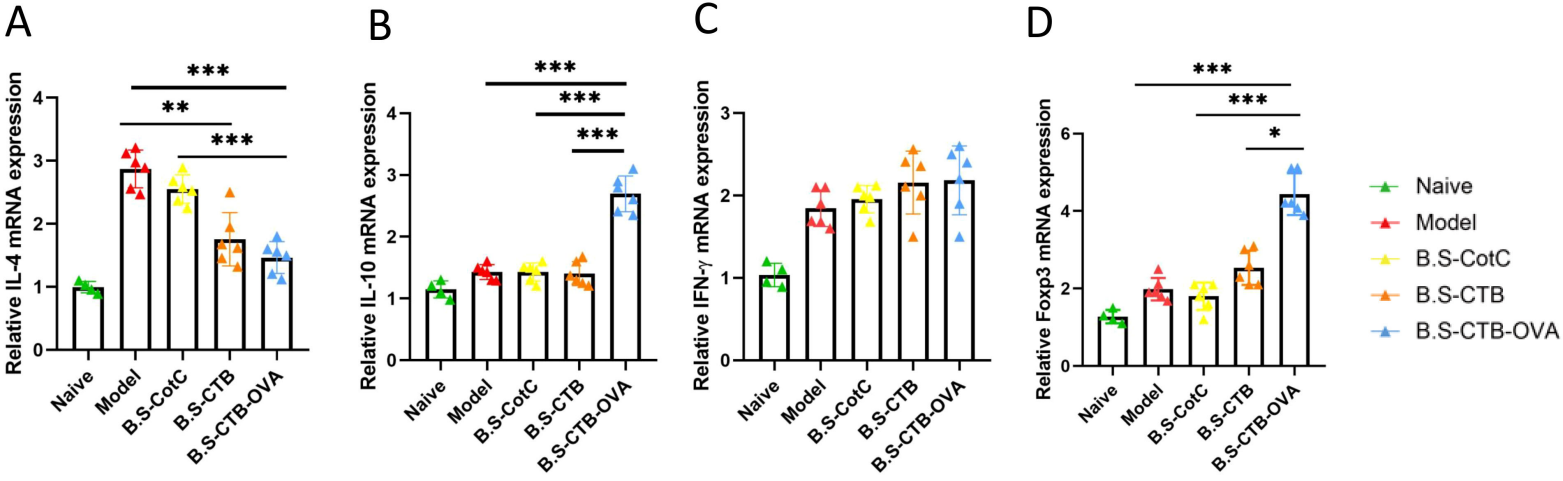
Oral administration of B.s-CotC-CTB-OVA spores modulates immune-related mRNA expression in jejunal tissues. Jejunal mRNA expression levels of IL-4, IL-10, IFN-γ, and Foxp3 were measured by qPCR. Relative expression levels were normalized to Gapdh and calculated using the 2^−ΔΔCt^ method. Data are shown as mean ± SD. Each symbol represents an individual mouse. *n* = 4 for the naïve group and *n* = 6 for the other groups. Statistical analyses were performed as described in the Methods section. *p < 0.05, **p < 0.01, ***p < 0.001.

To further evaluate local intestinal immune responses, we analyzed the mRNA expression levels of Foxp3, IL-4, IL-10, and IFN-γ in jejunal tissues by qPCR. Compared with the model group, oral administration of B.s-CotC-CTB-OVA spores significantly reduced jejunal IL-4 mRNA expression (*p* < 0.001), suggesting attenuation of local Th2-associated immune responses. IL-4 mRNA expression was also significantly lower in the *B.s*-CotC-CTB-OVA group than in the *B.s-*CotC group (*p* < 0.001). In contrast, IL-10 mRNA expression was significantly increased in the *B.s*-CotC-CTB-OVA group compared with the model, *B.s*-CotC, and *B.s*-CotC-CTB groups (all *p* < 0.001). Foxp3 mRNA expression was also significantly elevated in the *B.s*-CotC-CTB-OVA group compared with the model, *B.s*-CotC, and *B.s*-CotC-CTB groups (all *p* < 0.001), indicating an enhanced regulatory immune signature in jejunal tissues (**Figure 6**). Consistent with the splenocyte cytokine results, IFN-γ mRNA expression showed an increasing trend after *B.s*-CotC-CTB-OVA treatment, but the difference did not reach statistical significance. These findings suggest that *B.s*-CotC-CTB-OVA treatment is associated with a more regulatory intestinal immune environment, characterized by increased Foxp3 and IL-10 expression and reduced IL-4 expression.

### Microbiome profiles after oral administration of *B.s*-CotC-CTB-OVA spores

To determine whether oral administration of *B. subtilis* spores affected the gut microbiota before OVA sensitization, fecal samples were collected from five mice in each group after spore administration and subjected to 16S rRNA gene sequencing. We first compared microbial α- and β-diversity between mice receiving *B. subtilis* spores and naïve mice. The *B. subtilis* spore-treated mice showed a lower Shannon diversity index than naïve mice, although the difference was not statistically significant (Wilcoxon test, *p* > 0.05; **Figure 2A**). PCoA based on Bray–Curtis distances showed separation between the *B. subtilis* spore-treated and naïve groups, suggesting differences in overall microbial community structure (**Figure 2B**).

We next compared microbial diversity among the naïve, *B.s*-CotC, *B.s*-CotC-CTB, and *B.s*-CotC-CTB-OVA groups. No significant differences in α-diversity were observed among these groups (**Figure 2C**). PCoA revealed partially separated clusters with some overlap, indicating that different spore treatments were associated with modest changes in fecal microbiota composition (**Figure 2D**). Taxonomic composition at the family and genus levels is shown in **Figures 2E, F**. At the taxonomic level, oral administration of *B. subtilis* spores was associated with a significantly increased relative abundance of *Muribaculaceae* and a significantly decreased relative abundance of *Alistipes* compared with naïve mice (Wilcoxon test, *p* < 0.05; **Figures 2G, H**). When individual spore treatment groups were compared, the *B.s*-CotC-CTB-OVA group showed a trend toward decreased *Desulfovibrio* and increased *Odoribacter* relative abundance; however, these differences did not reach statistical significance (Wilcoxon test, *p* > 0.05; **Figures 2J, K**). No significant difference in *Lactobacillus* relative abundance was observed among the naïve, *B.s*-CotC, *B.s*-CotC-CTB, and *B.s*-CotC-CTB-OVA groups (**Figure 2I**). These findings indicate that oral *B. subtilis* spore administration is associated with changes in fecal microbiota composition, although treatment-specific differences among recombinant spore groups should be interpreted cautiously.

### FMT partially reproduces the reduction in serum OVA-specific IgE induced by *B.s*-CotC-CTB-OVA treatment

**Figure 7:**
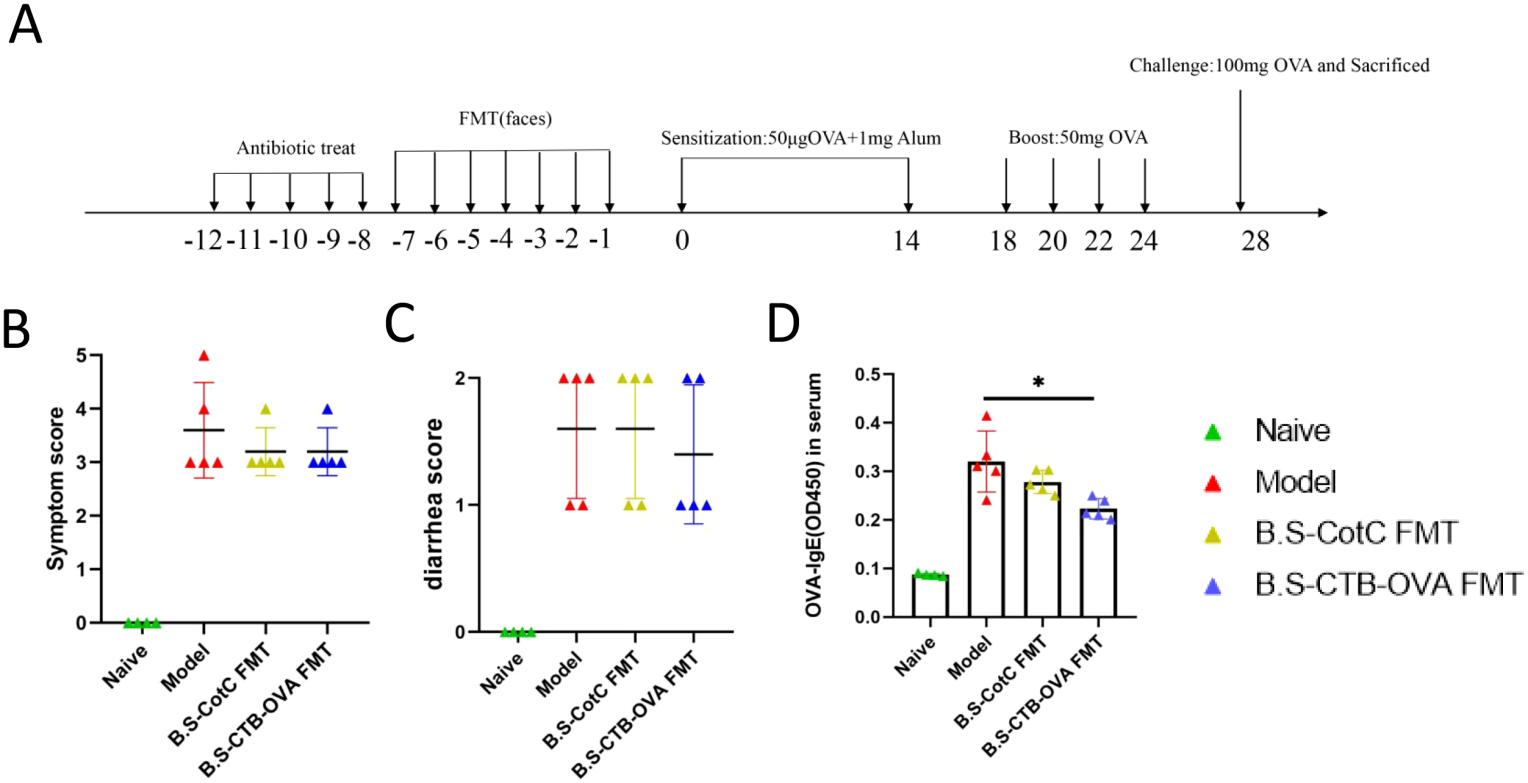
**(A)** Experimental protocol for antibiotics treatment, fecal microbiota transplant (FMT) from mice oral gavage of *B.s*-CotC*, B.s*-CotC-CTB-OVA, and naïve mice and the establishment of OVA food allergy mouse model. **(B)** Thirty minutes after challenge, the symptom scores of mice were observed and scored. **(C)** Thirty minutes after challenge, the diarrhea of mice was observed and scored. **(D)** Serum OVA-specific IgE at 60 min after the challenge on day 28. Data are shown as mean ± SD. Each symbol represents an individual mouse. *n* = 4 for the naïve group and *n* = 5 for the model, *B.s*-CotC, and B.s-CotC-CTB-OVA groups. Statistical analyses were performed as described in the Methods section. *p < 0.05, **p < 0.01, ***p < 0.001.

To investigate whether gut microbiota changes contributed to the protective effects of B.s-CotC-CTB-OVA, recipient mice were treated with a combination of antibiotics, including vancomycin, ampicillin, and metronidazole, from day −12 to day −8. The mice then received fecal microbiota transplantation from donor mice that had been orally administered *B.s*-CotC, *B.s*-CotC-CTB-OVA, or PBS. Recipient mice were subsequently sensitized and challenged with OVA according to the experimental protocol shown in **Figure 7A**.

After OVA challenge, mice receiving fecal microbiota from *B.s*-CotC-CTB-OVA-treated donors showed significantly reduced serum OVA-specific IgE levels compared with the corresponding control group (**Figure 7D**). However, no significant differences were observed in allergic symptom scores or diarrhea scores among the FMT groups (**Figures 7B, C**). These findings suggest that microbiota alterations induced by *B.s*-CotC-CTB-OVA treatment may partially contribute to the reduction in OVA-specific IgE, but are not sufficient to fully reproduce the clinical protection observed after direct *B.s*-CotC-CTB-OVA administration.

## Discussion

Early-life exposure to low levels of food antigens has been proposed as a potential strategy to promote immune tolerance and reduce the risk of allergic disease development in susceptible individuals. Previous studies have suggested that early exposure to allergens or microbial-derived signals, such as bacterial endotoxin, can reduce subsequent allergen-induced sensitization and allergic airway inflammation in mice (7, 25–27). However, the use of pre-immunization exposure to potential allergens as a primary prevention strategy for allergic diseases remains a field of active investigation and debate. Based on this concept, we developed a novel OIT strategy using *B. subtilis* spores as a delivery vehicle for a recombinant fusion protein composed of the main protein of egg white (OVA) (28) and the mucosal adjuvant CTB (*B.s*-CotC-CTB-OVA spores) to induce OVA immune tolerance in mice. CTB, a non-toxic subunit of the *V. cholerae* holotoxin, has been well documented to enhance tolerance to co-administered mucosal antigens (29). The utilization of *B. subtilis* spores exhibit strong resistance to gastric degradation, allowing the fusion protein to remain stable during gastrointestinal transit—making them a promising platform for oral antigen delivery (17, 30). Moreover, *B. subtilis* spores as a foreign bacterium of the gastrointestinal mucosal microbiome under gastric degradation can provide consistent low-level stable proteins to the individual, which make it an ideal vaccination delivery vehicle (11, 31, 32). Previous studies have proved that *B. subtilis* spores can display fusion proteins on their surface without compromising the structural integrity or immunogenic properties of the spores’ coat (16, 17).

Food allergy is typically characterized by gastrointestinal symptoms such as diarrhea, elevated serum IgE levels, and an increased number of eosinophils (33, 34). In the present study, oral administration of *B.s*-CotC-CTB-OVA spores before OVA sensitization increased fecal OVA-specific sIgA production and alleviated OVA-induced allergic responses in mice. Compared with the model and *B.s*-CotC groups, mice receiving *B.s*-CotC-CTB-OVA spores showed reduced allergic symptom severity, lower serum OVA-specific IgE levels, and decreased splenic IgE-positive cell frequencies. These findings suggest that *B.s*-CotC-CTB-OVA spores successfully delivered OVA-associated antigenic signals to the intestinal mucosa and promoted an antigen-specific mucosal immune response before allergic sensitization. Although *B.s*-CotC-CTB-OVA treatment also reduced diarrhea scores compared with the model group, no significant difference in diarrhea scores was observed between the *B.s*-CotC and *B.s*-CotC-CTB-OVA groups. Thus, the protective effect of *B.s*-CotC-CTB-OVA was more evident in overall allergic symptom severity than in diarrhea-related outcomes.

IgE-mediated food allergy is associated with an aberrant and hyperactive Th2 adaptive immune response to protein antigens (35, 36). In this study, *B.s*-CotC-CTB-OVA treatment reduced OVA-specific IgE and IgG1 levels, together with decreased IL-4 production, suggesting attenuation of Th2-associated allergic responses. Interestingly, OVA-specific IgG2a was also reduced, while IFN-γ showed only a non-significant increasing trend. Therefore, the effect of B.s-CotC-CTB-OVA cannot be simply interpreted as a classical shift from a Th2-dominant response toward a Th1-dominant response. Instead, the concurrent reduction of multiple OVA-specific antibody isotypes suggests that *B.s*-CotC-CTB-OVA may induce a broader antigen-specific regulatory or tolerogenic state.

Tregs play a critical role in maintaining immune tolerance and preventing excessive immune responses to harmless antigens in individuals (37, 38). They exert immunosuppressive effects through multiple mechanisms, including secretion of inhibitory cytokines such as IL-10 and TGF-β, suppression of effector T-cell activity, and modulation of antigen-presenting cell function (39, 40). In the present study, *B.s*-CotC-CTB-OVA treatment increased IL-10 production and enhanced splenic Treg frequencies, suggesting the involvement of regulatory immune responses. In addition to these systemic changes, *B.s*-CotC-CTB-OVA treatment increased jejunal Foxp3 and IL-10 mRNA expression while reducing IL-4 mRNA expression, indicating a more regulatory immune signature in intestinal tissues. Nevertheless, because Treg populations were not directly analyzed in gut-associated lymphoid tissues or intestinal lamina propria by flow cytometry, increased jejunal Foxp3 expression should be interpreted as evidence of a regulatory immune signature rather than direct evidence of intestinal Treg expansion. Although increased splenic Treg frequencies and elevated jejunal Foxp3 and IL-10 expression suggest enhanced regulatory immune responses, the present study did not directly assess intestinal Treg expansion or effector T-cell suppression at the cellular level. Future studies analyzing intestinal Treg and effector T-cell subsets in MLNs and lamina propria will be important to clarify whether *B.s*-CotC-CTB-OVA preferentially expands Tregs, suppresses effector T cells, or both.

Recent research has highlighted the crucial role of the human microbiome, particularly gut microbiome, in the development and onset of food allergy (41, 42). Alterations in the composition and diversity of microbial communities have been associated with abnormal immune responses to food antigens (9). In this study, 16S rRNA gene sequencing showed that oral administration of *B. subtilis* spores was associated with changes in fecal microbiota composition before OVA sensitization. *B. subtilis* spore administration was associated with increased relative abundance of *Muribaculaceae* and decreased relative abundance of *Alistipes.* Previous studies have suggested that elevated levels of *Alistipes* are associated with intestinal disorders/diseases including inflammatory bowel disease (IBD) and chronic kidney disease (43, 44). *Alistipes* can metabolize 5-hydroxyindole-3-acetic acid (5HIAA) (45). Oral gavage of 5HIAA has proved that it can potently mitigate DSS-induced colitis in mice. When individual recombinant spore groups were compared, *B.s*-CotC-CTB-OVA showed trends toward increased *Odoribacter* and decreased *Desulfovibrio*; *Odoribacter* has proved to be an important genus for maintaining intestinal homeostasis and shows high percentage in IBD (46). However, these differences did not reach statistical significance. Therefore, these taxa are reported as descriptive findings only, as the observed differences did not reach statistical significance and cannot be used to infer a mechanistic role in immune tolerance. Overall, the microbiome data suggest that *B. subtilis* spore administration is associated with alterations in gut microbial composition, but the taxonomic changes alone do not establish a causal microbiota-mediated mechanism of immune tolerance.

FMT has emerged as a novel approach for investigating the causal role of gut microbiome in health and disease (47, 48). FMT was performed to further explore whether microbiota alterations contributed to the protective phenotype. Recipient mice receiving fecal microbiota from *B.s*-CotC-CTB-OVA-treated donors showed reduced serum OVA-specific IgE levels after OVA challenge. However, FMT did not significantly improve allergic symptom scores or diarrhea scores. These results suggest that gut microbiota alterations may partially contribute to the immunological effects of *B.s*-CotC-CTB-OVA, particularly the reduction in OVA-specific IgE, but are not sufficient to fully reproduce the clinical protection observed after direct *B.s*-CotC-CTB-OVA administration. This difference may be related to the duration of treatment, the efficiency of microbiota engraftment, the absence of continuous antigen delivery, or the incomplete transfer of relevant microbial functions. Nonetheless, the relationship between the microbiome and disease remains a classic chicken-and-egg dilemma. It is still unclear whether alterations in the microbiome drive the alleviation of allergic symptoms, or whether the relief of allergy itself induces changes in the microbiome (49–51). Further research is needed to elucidate this relationship.

There are several limitations in this study. First, although splenic Tregs and jejunal immune-related mRNA expression were assessed, we did not directly examine Treg populations or effector T-cell subsets in gut-associated lymphoid tissues, intestinal lamina propria, or mesenteric lymph nodes by flow cytometry. Therefore, the precise cellular mechanisms underlying mucosal immune regulation remain to be clarified. Second, although *B.s*-CotC-CTB-OVA reduced OVA-specific IgE, IgG1, and IgG2a, additional Ig subclasses were not measured. Further studies assessing broader antibody profiles would help determine whether *B.s*-CotC-CTB-OVA induces generalized suppression of antigen-specific humoral responses. Third, 16S rRNA gene sequencing provides taxonomic information and should be interpreted as associative rather than mechanistic. Functional microbiome analyses, such as metabolomic profiling, short-chain fatty acid quantification, or predictive pathway analysis, were not performed in the present study. Therefore, the microbial metabolites or functional pathways involved in the observed phenotype remain unknown. Fourth, although mice used for microbiome analysis were age- and sex-matched and maintained under the same specific pathogen-free conditions, littermate effects and co-housing across treatment groups were not specifically controlled. Thus, cage-associated microbiota variation may have contributed to the observed microbiome differences. Finally, we did not determine the long-term duration of protection after *B.s*-CotC-CTB-OVA administration, nor did we evaluate its therapeutic effect after allergic sensitization had already been established.

In conclusion, oral administration of *B.s*-CotC-CTB-OVA spores before OVA sensitization alleviated OVA-induced allergic responses in mice. This effect was associated with increased fecal OVA-specific sIgA, reduced OVA-specific IgE and IgG1 responses, decreased allergic effector cell frequencies, increased IL-10 production, enhanced splenic Treg frequencies, and increased jejunal Foxp3 and IL-10 expression. *B. subtilis* spore administration was also associated with changes in fecal microbiota composition, for example, *Muribaculaceae* and *Alistipes*, and FMT partially reproduced the reduction in serum OVA-specific IgE. Together, these findings suggest that *B.s*-CotC-CTB-OVA spores may promote antigen-specific immune regulation and partially modulate gut microbiota-associated immune responses, supporting their potential as an oral strategy for food allergy prevention.

## Data Availability

The original contributions presented in the study are included in the article/**Supplementary Materials**, further inquiries can be directed to the corresponding authors.

## Supplementary material

Supplementary Figure 1 Strategy for construction of CotC-CTB-OVA gene fusions. OVA (1169 bp) was cloned in frame to the 5′ end of CTB in the pET-28a-CTB plasmid, yielding plasmid pET-28a-CTB-OVA, followed by CTB-OVA (1482 bp) that was cloned to the 3′ end of CotC in the pUS186-CotC plasmid, yielding plasmid pUS186-CotC-CTB-OVA.

Supplementary Figure 2 Representative stool appearances corresponding to diarrhea scores in mice: **(A)** score 0 – normal stool; **(B)** score 1 – soft stool; **(C)** score 2 – loose or liquid stool.

Supplementary Figure 3 Representative gating strategies for splenic eosinophils. **(A)** Gating strategy for splenic eosinophils. Debris was first excluded based on FSC-A and SSC-A characteristics. CD45^+^PI^-^ live leukocytes were then selected, and eosinophils were identified as CD11b^+^Siglec-F^+^ cells within the CD45^+^PI^-^ live-cell gate.

Supplementary Figure 4 Representative gating strategies for splenic eosinophils, mast cells, and regulatory T cells. Gating strategy for splenic mast cells. After debris exclusion based on FSC-A and SSC-A characteristics, single cells were selected using FSC-A and SSC-H. CD45^+^PI^-^ live leukocytes were then gated, and mast cells were identified as c-kit^+^IgE^+^ cells within the CD45^+^PI^-^ live single-cell gate. Percentages shown in the corresponding bar graphs represent frequencies within the indicated parent gates rather than percentages of total splenocytes.

Supplementary Figure 5 Representative gating strategies for splenic eosinophils, mast cells, and regulatory T cells. Gating strategy for splenic regulatory T cells. Lymphocytes were first gated based on FSC-A and SSC-A characteristics. CD4^+^ T cells were then selected, followed by exclusion of dead cells using Zombie staining. Regulatory T cells were identified as CD25^+^Foxp3^+^ cells within the CD4^+^ T-cell gate. Percentages shown in the corresponding bar graphs represent frequencies within the indicated parent gates rather than percentages of total splenocytes.
